# Effects of post-match foam rolling, static stretching, and passive rest on acute cardiac-autonomic, hemodynamic, and neuromuscular recovery in national wrestlers

**DOI:** 10.1186/s13102-026-01646-4

**Published:** 2026-03-06

**Authors:** Ali Kamil Güngör, Hüseyin Topçu, Yahya Yildirim, Andrew Flatt

**Affiliations:** 1 Faculty of Sport Sciences, Department of Coaching Education, Bursa Uludağ University, Bursa, 16000 Turkey; 2 Faculty of Sport Sciences, Department of Physical Education and Sport, Bursa Uludağ University, Bursa, 16000 Turkey; 3 Georgia Southern University, Health Sciences & Kinesiology, Savannah, Georgia 30460 USA

**Keywords:** Wrestling, Recovery methods, Anaerobic power, Heart rate variability, Blood pressure

## Abstract

Practical strategies that facilitate rapid restoration of cardiovascular and neuromuscular function are needed to support the short recovery window between matches in high-level wrestling tournaments. This study compared the effects of post-match foam rolling (FR), static stretching (SS), and passive rest (PR) on cardiac-autonomic, hemodynamic, and neuromuscular performance markers in national-level wrestlers. Sixteen male wrestlers (weight class range: 57–92 kg, ≥ 5 years of wrestling experience) participated in this randomized crossover study. Recovery interventions were performed on separate days immediately following a maximal-intensity match simulation. Heart rate variability (HRV), brachial blood pressure (BP), and countermovement jump (CMJ) parameters were measured pre-match, post-match, immediately post-recovery, and 10 min post-recovery. Across all recovery methods, HRV parameters showed progressive but incomplete recovery following post-match reductions (*p* < 0.05), while transient post-match BP increases were followed by a hypotensive response (*p* < 0.05). CMJ parameters were reduced at all post-match time points, with the greatest impairment observed immediately post-recovery (*p* < 0.05). Although no condition × time interactions were observed (*p* > 0.05), Hedges’ *g* effect sizes showed that at 10 min post-recovery, HRV parameters were higher for FR (*g* = 0.62–0.95), and to a lesser extent for SS (g = 0.54–0.69) versus PR. Mean arterial pressure was lower for FR versus PR (*g* = -0.69) at the same time point. While effect sizes suggest modest HRV recovery benefits favoring FR with neutral CMJ effects, the lack of statistical significance provides no conclusive evidence to recommend these modalities over PR for post-match recovery.

**Clinical trial number**

Trial registration: NCT07305506 (Registered date: 12 December 2025). This study was retrospectively registered on https://clinicaltrials.gov/study/NCT07305506.

**Supplementary Information:**

The online version contains supplementary material available at 10.1186/s13102-026-01646-4.

## Introduction

Freestyle wrestling is a high-intensity combat sport characterized by repeated bouts of explosive movements and rapid counterattacks. Matches typically consist of two 3-min periods separated by a 30-s rest, totaling 6 min of intense physical engagement [1]. Wrestling can elevate heart rate above 90% of maximum and blood lactate levels beyond 10 mmol/L [2], reflecting high anaerobic glycolytic demand, while the aerobic system facilitates recovery [3]. The ability to generate and sustain muscular power in extremities is a critical determinant of performance [3, 4]. However, in tournament settings, performance across multiple matches depends not only on physical capacities but also on the effectiveness of recovery between bouts. At elite competitions such as the European or World Championships, wrestlers compete in up to 4–5 matches in a single day, with rest periods lasting approximately 90 min. Despite intense physiological demands and congested match schedules, few studies have examined short-term recovery strategies in high-level wrestlers. Thus, an investigation into practical methods that may expedite recovery within this narrow window would address an important research gap in competitive wrestling.

Sympathetic activation predominates during intense activities such as wrestling, which contributes to increases in cardiac output, blood pressure, respiration, and glucose mobilization to meet high cardiometabolic and psychophysiological demands [5]. Following a match, parasympathetic reactivation counters sympathoexcitation and supports cardiovascular recovery [5]. Heart rate variability (HRV) reflects beat-to-beat fluctuations in cardiac intervals and is commonly used to evaluate parasympathetic modulation at rest and post-exercise [5].Whereas HRV is used to reflect cardiac parasympathetic recovery, blood pressure (BP) recovery is relevant for evaluating hemodynamic and vascular restoration, and serves as a complementary marker of overall cardiovascular recovery [6, 7].

Strategies that accelerate post-match HRV recovery may confer performance benefits in subsequent bouts. For example, cold water immersion improved cardiac parasympathetic reactivation during recovery from a simulated rugby sevens match, followed by enhanced sympathetic drive during a subsequent intra-day match simulation [8]. This was reflected in higher mean and peak heart rate and blood lactate concentrations during repeated sprint testing (incorporated into the initial 2 min of each match simulation) relative to passive rest (PR) [8]. Notably, higher heart rate values during repeated sprint tests were moderately correlated with superior performance outcomes [8]. While these findings highlight the performance benefit of accelerating cardiac-parasympathetic reactivation, cold water immersion is impractical in wrestling tournament settings, and may adversely affect neuromuscular performance, such as the countermovement jump (CMJ), within very short recovery windows [9]. Given the importance of lower-body power in wrestling [4], alternative recovery modalities that support cardiovascular recovery without compromising neuromuscular function require investigation.

Foam rolling (FR) and static stretching (SS) are appealing post-match recovery modalities due to their low cost, accessibility, and ease of implementation during short recovery intervals. These modalities require no or minimal equipment and are familiar to many wrestlers. Evidence suggests that FR may enhance recovery by improving muscle function and cardiac-autonomic modulation [10–13], though findings are inconsistent [14–16]. For example, significant increases in HRV and reductions in diastolic BP have been observed following FR [13, 17], while others report no significant effects [18], particularly when FR is applied after exercise [14, 16]. Similarly, SS has been reported to improve HRV and lower BP in certain contexts [19–21], but concerns remain regarding its potential to impair neuromuscular performance, especially explosive power output [22–25]. Given inconsistent findings and limited research in wrestling-specific contexts, further investigation is warranted to determine whether FR or SS can effectively support cardiovascular recovery without impairing neuromuscular performance. Therefore, we aimed to compare the acute effects of post-match FR, SS, and PR on cardiac-autonomic, hemodynamic, and neuromuscular performance markers in national-level male wrestlers.

## Materials and methods

### Study design

**Fig. 1:**
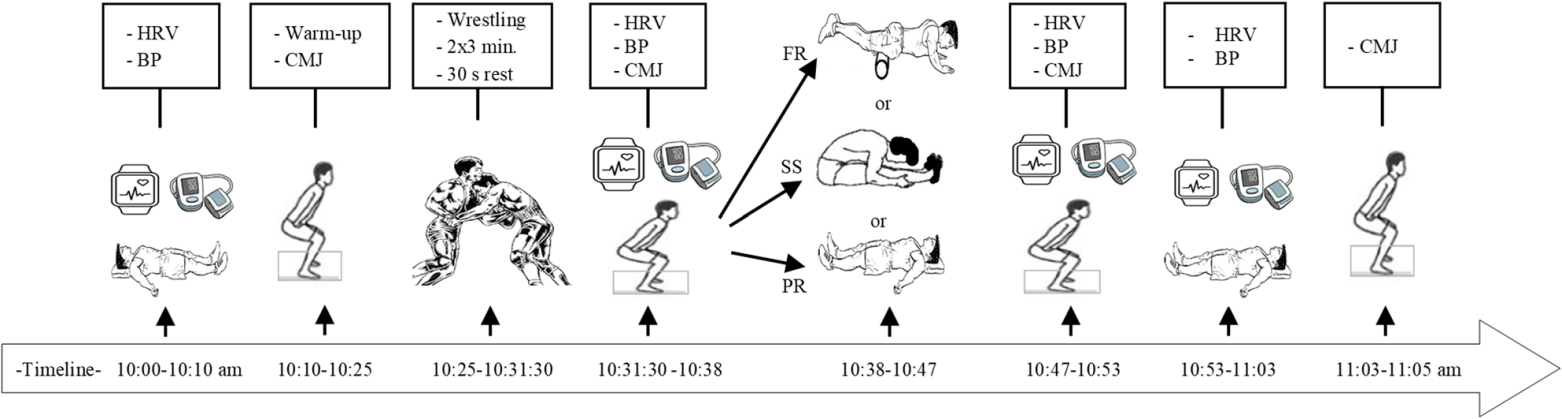
The implementation procedure and measurements of heart rate variability, blood pressure, and countermovement jump

This study employed a randomized controlled crossover design. To control potential order effects, the sequence of the interventions was balanced using a Latin Square design. The randomization sequence was created using a computer-generated random number list. To ensure allocation concealment, the randomization process was managed by an independent researcher. Male national freestyle wrestlers competing in weight categories between 57 and 92 kg were randomly assigned to one of three recovery interventions following a maximal effort wrestling match: FR, SS, or PR. To prevent fatigue accumulation and carry-over effects, a minimum washout period of 72 h was mandated between sessions. All experimental sessions for each participant were completed within a three-week period. The first visit was dedicated to familiarizing subjects with the study procedures and recovery techniques, while the recovery protocols were administered during the second, third, and fourth visits. Measurements of HRV, BP, and countermovement jump (CMJ) were taken at four time points: pre-match, immediately post-match, immediately post-recovery (FR, SS, or PR), and again 10 min post-recovery period. This post-exercise time frame is consistent with previous studies examining post-training FR effects on HRV [16, 26], and was chosen to capture the acute, direct effects of each modality during the initial phase of post-match simulation recovery. Moreover, SS-related changes in CMJ are not expected to occur beyond 10 min with our selected SS protocol [27]. Environmental conditions were standardized to ensure consistency across sessions. The experimental protocol is visualized in Fig. 1.

### Subjects

Men’s national-level male wrestlers with at least five years of wrestling experience were voluntarily recruited from the Turkey Olympic Preparation Center (TOPC). The study included wrestlers competing in the World Wrestling Championship weight categories (57, 61, 65, 70, 74, 79, 86, and 92 kg, one pair of wrestlers in each category). Inclusion criteria were: (i) being freestyle wrestlers and 18–24 years old, (ii) holding an active wrestling license for at least 5 years, (iii) possessing at least a C-level National Athlete Certificate (having represented Turkey in at least one international senior wrestling tournament organized by United World Wrestling), v) having trained regularly for the last 12 months, and vii) having had no injuries in the past 12 months. Exclusion criteria were: (i) having any cardiovascular health issues, (ii) using medications or substances affecting the respiratory or cardiovascular system, (iii) using ergogenic dietary supplements (e.g., creatine, caffeine), (iv) having a resting systolic blood pressure (SBP) ≥ 140 mmHg and diastolic blood pressure (DBP) ≥ 90 mmHg [28]. According to the participant classification framework proposed by McKay et al. [29], the wrestlers in the present study would be categorized as Tier 4 (Elite/International Level), given their status as national team members preparing at an Olympic center with prior representation of Turkey in senior international UWW tournaments.

Written informed consent was obtained after subjects were thoroughly informed about the study procedures, requirements, potential benefits, and associated risks. The study followed the principles outlined in the Declaration of Helsinki and was approved by the local Research Ethics Committee (Approval code: 2023-21/18). The reporting of this study adheres to the CONSORT guidelines for reporting clinical trials.

A priori power analysis was conducted using G Power software (version 3.1.9.7) for the F-test family (repeated measures ANOVA, within-subject factors) to determine the required sample size. We specifically referenced [16], which used a sample of 16 participants and reported a very large effect size (f = 1.08, *n* = 9). Accordingly, we selected an effect size of f = 0.35 to avoid overestimation. The power analysis indicated that a sample size of 13 subjects across three sessions with four measures would achieve a statistical power of β = 0.80 at an alpha level of α = 0.05. To account for potential dropouts, we recruited 16 participants, which provided sufficient power (> 80%) to detect the specified effect size [30]. There were no dropouts or exclusions after randomization.

### Familiarization session

A dedicated familiarization session was conducted approximately one week prior to the first experimental trial. At the beginning of this visit, after obtaining written informed consent, subjects’ anthropometric characteristics were assessed. Body weight (kg), fat (%), and body mass index (BMI, kg/m²) were assessed using a body composition analyzer (Tanita Model BF-350; Tanita Corp., Tokyo, Japan) with subjects wearing minimal clothing (shorts and t-shirt). Subjects’ gender, age, and height were entered into the system, and measurements were recorded using the “athletic mode.”

Following the assessments, subjects were familiarized with the testing procedures and recovery interventions to minimize learning effects. They performed multiple practice trials of the CMJ until a consistent technique was achieved. Additionally, subjects practiced the FR and SS protocols under the supervision of the researchers. Particular attention was given to the correct tempo (2 s up, 2 s down using a metronome for FR) and the specific techniques required for each muscle group, ensuring they could replicate the protocols accurately during the experimental sessions.

### Training interventions

Recruitment and data collection took place between January 2024 and February 2024. Wrestling matches were consistently performed in the morning between 10:00 and 11:05 to control circadian variation effects. The gym temperature was maintained between 22 and 24 °C, and humidity was set between 33 and 45%. A standardized 15-min wrestling-specific warm-up was completed for each session under the supervision of a coach. Afterwards, wrestlers in the same weight categories competed in a maximal effort wrestling match following official match protocols (2 × 3-min rounds, 30 s rest in between) [31]. Subsequently, wrestlers completed one of the recovery interventions (PR, SS, or FR, Table 1). PR was performed by lying still in a supine position. Each recovery protocol lasted 9 min. To ensure the fidelity of the interventions, all recovery sessions (FR, SS, and PR) were supervised by the investigator. They ensured that subjects adhered strictly to the specific techniques, durations, and tempo (using a metronome for FR) as defined in the protocol. No adverse events, injuries, or unintended side effects were reported by the subjects during the wrestling matches, recovery interventions (FR, SS, PR), or testing procedures. Subjects were instructed to abstain from caffeine, alcohol, and intense exercise for at least 24 h, and refrain from food consumption for 3 h, and liquid intake for 1 h before matches [32].

**Table 1:** Recovery intervention protocols for foam rolling and static stretching

Foam Roller			Static Stretching		
Exercises	Set x duration	Rest	Exercises	Set x duration	Rest
Gastrocnemius and soleus roll	2 × 30 s	30 s	Gastrocnemius and soleus stretch	2 × 30 s	30 s
Anterior lower-leg muscles roll	2 × 30 s	30 s	Anterior lower-leg muscles stretch	2 × 30 s	30 s
Hamstrings roll	2 × 30 s	30 s	Hamstrings stretch	2 × 30 s	30 s
Quadriceps femoris roll	2 × 30 s	30 s	Quadriceps femoris stretch	2 × 30 s	30 s
Gluteal muscles roll	2 × 30 s	30 s	Gluteal muscles stretch	2 × 30 s	30 s
Thoracolumbar musculature roll	2 × 30 s	30 s	Thoracolumbar musculature stretch	2 × 30 s	30 s

### Foam roller procedure

Subjects performed the FR intervention using a uniform polystyrene high-density roller (6 inches in diameter and 36 inches in length) from Trigger Point Technologies (Austin, Texas, USA). The protocol targeted the calf muscles (gastrocnemius and soleus), anterior lower-leg muscles, quadriceps femoris, hamstrings, gluteal muscles, and thoracolumbar musculature. Subjects performed six different exercises that required them to work the entire surface area by rolling the targeted muscle groups back and forth on the foam roller between the origin and insertion of muscles. Each repetition consisted of moving the target tissue along the roller in a smooth motion for 2 s down and 2 s up, set by a metronome. Constant feedback was given by the researcher to maintain correct form and rhythm. The execution procedure of the FR exercises was adapted from Lastova et al. [11].

### Static stretching procedure

The static stretching protocol included six exercises targeting the same muscle groups as FR and performed in the same order. Active SS involved elongating the muscle to the point of discomfort and holding the position without movement. All exercises were performed bilaterally, with both extremities stretched simultaneously, without partner assistance. Each stretch was held for 30 s and repeated for two sets, with 30 s of rest between sets and exercises.

### Measurement procedures

#### Heart rate variability assessment 

R-R intervals were recorded using a V800 HR monitor and H10 Bluetooth chest strap (Polar Electro OY, Kempele, Finland) [33]. Time points for HRV analysis were as follows: pre-match before the warm-up (5-min sample after a 5-min stabilization), immediately post-match (2-min sample following a 2-min stabilization), immediately post-recovery (2-min sample following a 2-min stabilization), and 10 min post-recovery (5-min sample after a 5-min stabilization) [34]. Measurements were performed in the supine position while subjects remained quiet, still, and breathed naturally. R-R interval data were subsequently transferred to a computer via the Polar Flow application for analysis. Kubios HRV software (Standard version 3.5.0, University of Kuopio, Kuopio, Finland) was used to calculate HRV parameters. The software automatically performed smoothness priors detrending procedures [35], removed noise, and applied artifact correction with a very low threshold (not exceeding 2% in the current sample). To estimate cardiac-autonomic modulation, the following time domain measurements were recorded: Mean-RR, which represents the average time interval between consecutive R peaks and is inversely related to heart rate, the root mean square of the successive differences of the RR intervals (RMSSD), which predominantly reflects parasympathetic modulation, and standard deviation of normal R-R intervals (SDNN), which reflects overall HRV and represents both sympathetic and parasympathetic influences [36].

#### Blood pressure assessment

Supine brachial BP measurements were taken to determine systolic, diastolic, and mean arterial pressure (SBP, DBP, and MAP, respectively) using an automatic oscillometric device (Omron M2 HEM-7121-E) with a measurement range of 10–290 mmHg and a resolution of 1 mmHg. The equipment was automatically calibrated before each use. Measurements were made on the left arm in accordance with the guidelines provided by the American Heart Association [37]. Measurements were taken immediately following R-R interval recordings at the same four time-points.

#### Muscle power assessment

The CMJ test was employed to assess the explosive strength of the lower extremity muscles by maximizing stretch-shortening cycle activity [38]. During the test, subjects were instructed to perform an explosive vertical jump on a platform. They were further instructed to maintain their hands on their hips, keep their knees fully extended, and self-select the depth of the CMJ before jumping vertically at maximum speed. Any technical deviation, such as removing the hands from the hips or pulling the knees up during the flight phase, was considered an error, and the test was repeated [39]. Each subject performed the test twice with an interval of 30 s, and the highest score was recorded. CMJ height was assessed using the Optojump Next system (Microgate, Bolzano, Italy), which detects flight time to calculate jump height. The Optojump system has been shown to be a valid and reliable tool for assessing jump performance [40]. Peak wattage was manually calculated using the Sayers equation [41], which estimates lower-limb power output from jump height and body mass during CMJ performance. The formula is as follows: “Peak Power (watt) = (60.7×jump height (cm)) + (45.3×body mass (kg)) – 2055.”

### Statistical analysis

The data were analyzed using SPSS version 28.0 (IBM Corp., Armonk, NY, USA). Descriptive statistics are reported as mean ± standard deviation. All subjects who completed all three experimental conditions were included in the analysis, with no missing data. The normality of all variables was assessed with the Shapiro–Wilk test. A two-way repeated measures ANOVA (3 sessions × 4 time points) was conducted to assess session, time, and interaction effects for HRV, BP, and CMJ variables. The significance level was set at *p* < 0.05. When appropriate, post-hoc multiple comparisons were conducted with Bonferroni adjustments. Effect size (ES) was calculated using partial eta squared (${n}_{p}^{2}$) (0.01, 0.06, and 0.14 indicating small, medium, and large effects, respectively [42]), from the repeated measures ANOVA. Standardized differences were also calculated for pairwise comparisons using Hedges’ *g* ES with 95% confidence intervals (CI), as recommended by Lakens [43]. Hedges’ *g* is bias-corrected and particularly appropriate when a sample size is < 20 [43]. This statistic enables quantification of the practical magnitude of differences between conditions in a scale-independent manner, facilitates meta-analytic comparisons across studies, and informs a priori power analyses for future research [43, 44]. Hedges’ *g* ES was deemed unclear if the 95% CI overlapped both substantially positive (0.20) and negative (-0.20) values [45].

## Results

The wrestlers had a mean age of 20.3 ± 2.1 years, body weight of 71.0 ± 10.8 kg, height of 173.8 ± 7.8 cm, body mass index of 23.4 ± 1.9 kg/m², body fat mass % 11.1 ± 2.6, and training experience of 9.3 ± 2.3 years.

### HRV data

**Fig. 2:**
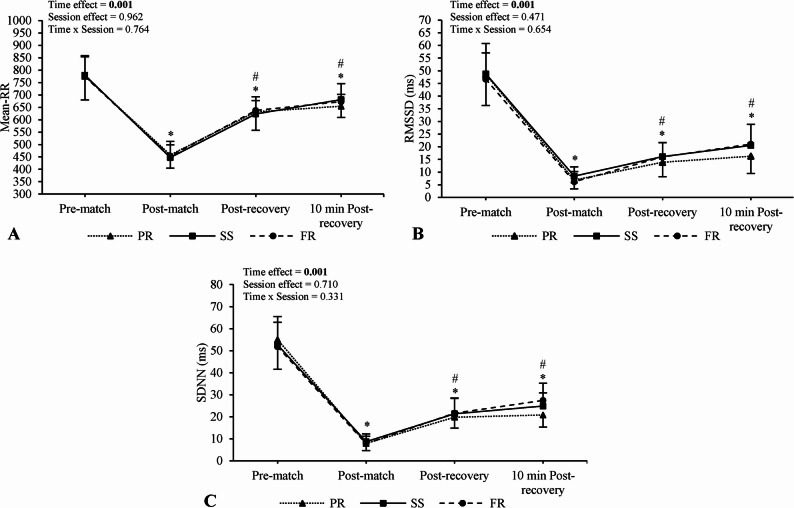
Comparison of heart rate variability parameters between sessions and across time values. Numerical values shown in the figure represent *p*-values. *Different from pre-match values (main time effect), ^#^different from post-match values (main time effect). RMSSD; the root of the mean of the square of the difference of the RR intervals; SDNN; standard deviation of normal R-R intervals, PR; passive recovery, SS; static stretching, FR; foam roller

No main effects of condition (Mean-RR, F = 0.039, *p* = 0.962, ${n}_{p}^{2}$ = 0.003; RMSSD, F = 0.773, *p* = 0.471, ${n}_{p}^{2}$ = 0.049; SDNN, F = 0.346, *p* = 0.710, ${n}_{p}^{2}$ = 0.023) or condition × time interactions were observed for any HRV parameter (Mean-RR, F = 0.556, *p* = 0.764, ${n}_{p}^{2}$ = 0.036; RMSSD, F = 0.695, *p* = 0.654, ${n}_{p}^{2}$ = 0.044; SDNN, F = 1.167, *p* = 0.331, ${n}_{p}^{2}$ = 0.072). Despite no significant interaction, standardized differences indicated higher values for SS (RMSSD: g = 0.54, 95% CI [–0.16, 1.25]; SDNN: g = 0.69, 95% CI [–0.02, 1.41]) and FR (RMSSD: g = 0.62, 95% CI [–0.09, 1.33]; SDNN: g = 0.95, 95% CI [0.22, 1.68]) versus PR at 10 min post-recovery. In contrast, between-condition standardized differences for Mean-RR were unclear (SS vs. PR: g = 0.45, 95% CI [–0.25, 1.16]; FR vs. PR: g = 0.31, 95% CI [–0.38, 1.01]). Post-hoc condition × time plots are presented in Fig. 2.

Main effects of time were observed for Mean-RR (F = 251.63, *p* < 0.001, ${n}_{p}^{2}$ = 0.94), RMSSD (F = 399.05, *p* < 0.001, ${n}_{p}^{2}$ = 0.96), and SDNN (F = 476.01, *p* < 0.001, ${n}_{p}^{2}$ = 0.97). Mean-RR was reduced post-match (*p* < 0.001; g = − 7.44, 95% CI [–9.39, − 5.49]), immediately post-recovery (*p* < 0.001; g = − 2.86, 95% CI [–3.84, − 1.87]) and 10 min post-recovery (*p* < 0.001; g = − 2.30, 95% CI [–3.20, − 1.41]) compared to pre-match. However, values increased immediately post-recovery (*p* < 0.001; g = 4.64, 95% CI [3.31, 5.97]) and 10 min post-recovery (*p* < 0.001; g = 6.75, 95% CI [4.96, 8.54]) compared to post-match.

Similar trends were observed for RMSSD and SDNN, with reductions post-match (RMSSD: g = − 8.00, 95% CI [–10.07, − 5.92]; SDNN: g = − 9.68, 95% CI [–12.15, − 7.21]), immediately post-recovery (RMSSD: g = − 5.80, 95% CI [–7.38, − 4.22]; SDNN: g = − 5.79, 95% CI [–7.37, − 4.21]), and 10 min post-recovery (RMSSD: g = − 5.38, 95% CI [–6.87, − 3.89]; SDNN: g = − 5.79, 95% CI [–7.37, − 4.21]) compared to pre-match (all *p* < 0.001). However, values increased immediately post-recovery (RMSSD: g = 2.27, 95% CI [1.38, 3.16]; SDNN: g = 3.26, 95% CI [2.21, 4.32]) and 10 min post-recovery (RMSSD: g = 3.89, 95% CI [2.71, 5.06]; SDNN: g = 5.39, 95% CI [3.93, 6.88]) compared to post-match (all *p* < 0.001).

### BP data

**Fig. 3:**
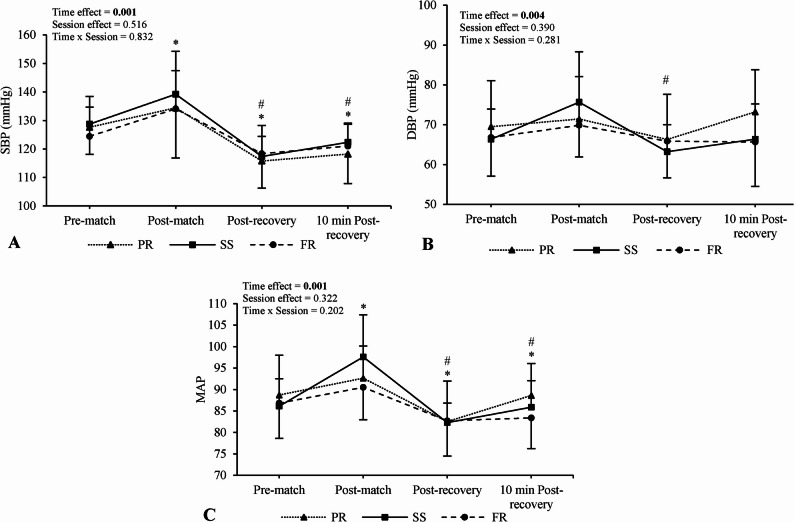
Comparison of blood pressure parameters between sessions and across time. Numerical values shown in the figure represent *p*-values. *Different from pre-match values (main time effect), ^#^different from post-match values (main time effect). SBP; systolic blood pressure, DBP; diastolic blood pressure, MAP; mean arterial pressure, PR; passive recovery, SS; static stretching, FR; foam roller

No main effects of condition (SBP, F = 0.679, *p* = 0.516, ${n}_{p}^{2}$= 0.050; DBP, F = 0.978, *p* = 0.390, ${n}_{p}^{2}$= 0.070; MAP, F = 1.151, *p* = 0.322, ${n}_{p}^{2}$= 0.081) or condition × time interactions were observed for any BP parameter (SBP, F = 0.466, *p* = 0.832, ${n}_{p}^{2}$ = 0.035; DBP, F = 1.270, *p* = 0.281, ${n}_{p}^{2}$= 0.089; MAP, F = 1.463, *p* = 0.202, ${n}_{p}^{2}$= 0.101). Additionally, all between-condition standardized differences for BP parameters at 10 min post-recovery were deemed unclear, except for MAP being lower for FR compared to PR (g = -0.69, 95% CI [-1.40, 0.02]). Post-hoc condition × time plots are presented in Fig. 3.

Main effects of time were observed for SBP (F = 32.55, *p* < 0.001, ${n}_{p}^{2}$ = 0.72), DBP (F = 5.12, *p* = 0.004, ${n}_{p}^{2}$ = 0.28), and MAP (F = 18.65, *p* < 0.001, ${n}_{p}^{2}$ = 0.59). SBP increased post-match compared to pre-match (*p* = 0.008; g = 1.16, 95% CI [0.41, 1.91]), but decreased immediately post-recovery (*p* < 0.001; g = − 1.60, 95% CI [–2.39, − 0.80]) and 10 min post-recovery (*p* = 0.002; g = − 0.95, 95% CI [–1.68, − 0.22]) compared to pre-match, as well as versus post-match (*p* < 0.001; g = − 2.65, 95% CI [–3.60, − 1.70]; *p* < 0.001; g = − 2.01, 95% CI [–2.86, − 1.16], respectively). 

DBP decreased immediately post-recovery compared to post-match (*p* < 0.001; g = − 1.14, 95% CI [–1.88, − 0.39]). MAP increased post-match (*p* = 0.002; g = 1.23, 95% CI [0.47, 1.98]) and decreased immediately post-recovery (*p* = 0.004; g = − 1.08, 95% CI [–1.82, − 0.33,]) compared to pre-match, with further decreases immediately post-recovery (*p* < 0.001; g = − 2.03, 95% CI [–2.88, − 1.17]) and 10 min post-recovery compared to post-match (*p* = 0.009; g = − 1.27, 95% CI [–2.03, − 0.51]).

### CMJ data

**Fig. 4:**
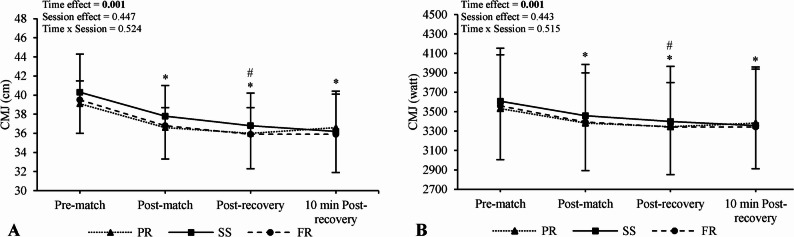
Comparison of countermovement jump parameters between sessions and across time. Numerical values shown in the figure represent *p*-values. *Different from pre-match values (main time effect), ^#^different from post-match values (main time effect). CMJ; countermovement jump, PR; passive recovery, SS; static stretching, FR; foam roller

No main effects of condition (CMJ_cm_, F = 0.830, *p* = 0.447, ${n}_{p}^{2}$= 0.060; CMJ_watt_, F = 0.840, *p* = 0.443, ${n}_{p}^{2}$= 0.061) or condition × time interactions were observed for any CMJ parameter (CMJ_cm_, F = 0.865, *p* = 0.524, ${n}_{p}^{2}$= 0.062; CMJ_watt_, F = 0.878, *p* = 0.515, ${n}_{p}^{2}$= 0.063), and standardized differences were all unclear. Post-hoc condition × time plots are presented in Fig. 4.

Main effects of time were observed for CMJ_cm_ (F = 47.04, *p* < 0.001, ${n}_{p}^{2}$= 0.78) and CMJ_watt_ (F = 47.06, *p* < 0.001, ${n}_{p}^{2}$= 0.78). CMJ values were reduced post-match (*p* < 0.001; g = − 0.87, 95% CI [–1.60, − 0.15]; *p* < 0.001; g = − 0.28, 95% CI [–0.97, 0.42,], respectively), immediately post-recovery (*p* < 0.001; g = − 1.10, 95% CI [–1.84, − 0.35]; *p* < 0.001; g = − 0.36, 95% CI [–1.06, 0.34], respectively) and 10 min post-recovery (*p* < 0.001; g = − 1.01, 95% CI [–1.75, − 0.28]; *p* < 0.001; g = − 0.36, 95% CI [–1.06, 0.33], respectively) compared to pre-match. Additionally, CMJ values were reduced post-recovery compared to post-match (*p* = 0.007; g = − 0.29, 95% CI [–0.98, 0.41]; *p* = 0.008; g = − 0.09, 95% CI [–0.78, 0.60], respectively). All standardized differences were deemed unclear for both parameters despite statistically significant effects (*p* < 0.05).

## Discussion

The purpose of this study was to compare the acute effects of post-match FR, SS, and PR on cardiac-autonomic, hemodynamic, and neuromuscular performance markers in national-level wrestlers. Across all recovery methods, cardiac-autonomic parameters showed progressive but incomplete recovery post-match, while hypotensive effects were observed following transient post-match increases in BP. Neuromuscular performance, as assessed by CMJ, was reduced at all post-match time points, with the greatest impairment observed immediately post-recovery. Although no statistically significant condition × time interactions were observed, Hedges’ *g* analyses at 10 min post-recovery revealed the largest standardized differences for FR versus PR in RMSSD and SDNN, followed by smaller differences for SS versus PR in RMSSD and SDNN. Additionally, MAP was lower in FR than in PR at the same time point. These magnitude-based findings suggest possible modest practical benefits of FR, and to a lesser extent SS, on cardiac-autonomic and hemodynamic recovery relative to passive rest, without negatively impacting neuromuscular performance in national-level wrestlers. However, the absence of statistically significant condition × time interactions underscores that these observations are exploratory and require confirmation through additional research.

Cardiac-autonomic parameters were significantly reduced post-match, then partially and progressively increased post-recovery, regardless of condition. Nearly abolished post-match HRV reflects the very intense anaerobic workload of elite-level wrestling, which generates blood lactate levels > 10 mmol/L and heart rates > 90% of maximum [2]. This acute attenuation of HRV involves withdrawn parasympathetic modulation and increased sympathetic activation, likely driven by factors such as competitive stress and arousal, increased cardiac output and respiration to meet physical demands, and metaboreflex stimulation from elevations in lactate and other metabolites [5]. Restoration of HRV to baseline following maximal intensity anaerobic exercise can take ≥ 24 h [5]. Therefore, incomplete recovery at 10 min post-recovery was expected. Consistent with our findings, Pernigoni et al. [16] found no significant difference in RMSSD between a FR and control group in female basketball players, neither immediately post-match (5-min recording) nor 24 h later. Similarly, D’Amico et al. [14] reported no significant differences in RMSSD immediately following a 40 × 15 sprint training session in healthy adults.

Although not statistically significant, magnitude-based analyses demonstrated that RMSSD and SDNN for both interventions were higher than control at 10 min post-recovery, with larger magnitudes observed for FR. Contrasting with previous findings [14, 16], Güngör et al. [13] demonstrated that post-exercise FR significantly accelerated post-submaximal exercise recovery of RMSSD and high frequency HRV versus control in amateur basketball players. Meanwhile, Lastova et al. [11] compared HRV responses to acute FR versus a time-matched non-exercise control, and reported significant increases in normalized high frequency HRV post-FR. The mechanisms underpinning potential FR-induced increases in HRV may involve stimulation of mechanoreceptors (e.g., Ruffini endings), leading to decreased sympathetic activation [46], as well as enhanced nitric oxide release via augmented blood flow, which has been shown to support lactate clearance [47] and promote parasympathetic reactivation [48, 49].

For SS, the literature is similarly mixed. While several studies [19, 50, 51] have reported increased, fewer have examined its effects on post-exercise recovery. Yıldırım et al. [21] found no significant differences in HRV indices following SS, dynamic stretching, and PR after simulated wrestling matches in 12 elite male wrestlers. Conversely, Pernigoni et al. [25] reported higher post-recovery natural log-transformed RMSSD values following SS compared to active recovery in male basketball players after a 90-min training session, though no control condition was included. The mechanisms by which acute SS may enhance resting HRV involve improved baroreflex sensitivity, reductions in vascular sympathetic activity, nitric oxide–mediated vasodilation, and activation of a relaxation response, which may reduce respiratory rate [52]. However, current and previous findings [21] suggest that the stimulatory effects of SS on HRV may be less pronounced when performed post-maximal exertion.

Hemodynamic parameters showed increased SBP and MAP post-match, followed by reductions through the recovery period relative to both pre- and post-match. In contrast, DBP showed a significant reduction only immediately post-recovery versus post-match. This hemodynamic pattern is consistent with the expected transient rise in post-exercise SBP due to increased cardiac output and sympathetic activation, followed by a hypotensive response driven by sustained vasodilation, sympathoinhibition, and lowered cardiac output [53]. Others have also reported no significant effects of recovery modalities on BP responses relative to control. For example, Petviset et al. [54] found no significant BP differences after 15 min of SS or yoga recovery following intense training in university-level soccer players. Additionally, Jafari et al. [55] found no BP differences in 16 elite wrestlers after four recovery methods: jogging, jogging with SS, jogging with dynamic stretching, and PR. However, our magnitude-based analysis revealed lower MAP for FR versus PR at 10 min post-recovery, coinciding with higher RMSSD and SDNN. This suggests potential concurrent effects of post-exercise FR on cardiac-autonomic and hemodynamic responses. Similar findings have been reported in an investigation that did not include an exercise bout. Significant reductions in SBP and DBP with concurrent increases in high frequency HRV, despite no change in HR, were observed following FR in habitual exercisers versus a control group during a 30-min recovery period [11]. Further supporting the BP-lowering effects of FR, Ketelhut et al. [56] reported decreases in SBP, DBP, and MAP after 30 min of FR in 29 recreationally active men. Mechanistically, FR may exert localized pressure on the fascia, potentially modulating vascular tone through reduced arterial stiffness and improved endothelial function [49].

Neither FR nor SS led to a meaningful restoration of CMJ parameters relative to PR. Rather, persistent decrements were observed across all recovery methods. These results agree with several prior investigations reporting limited effects of FR on jump performance [15, 16, 57–60]. Indeed, a recent meta-analysis by Wiewelhove et al. [27] concluded that post-exercise FR had a trivial effect on various markers of jump performance (− 0.2%, g = 0.06). Similarly, SS had neutral effects on CMJ performance in our study. Although SS can acutely impair explosive performance, our SS protocol (2 × 30 s per muscle group) was intentionally designed to not exceed established dose-response thresholds. For example, SS durations > 60 s reduce power-related tasks by -4.6%, whereas SS durations < 60 s minimally affect performance [61]. Decrements in explosiveness induced by SS are attributable to decreases in muscle-tendon stiffness and neuromuscular activation [62], as well as alterations in the viscoelastic properties of muscle fibers, which may affect the length-tension relationship and sarcomere kinetics [63]. Taken together, FR or SS performed for 2 × 30 s per muscle group had neutral effects on post-match CMJ outputs in national-level wrestlers.

This study has various limitations. First, blood biomarkers relevant to fatigue and recovery, such as blood lactate, were not monitored. Second, outcomes were tracked for only 10 min after the recovery interventions, which may not capture longer-term physiological effects. Additionally, performance was not assessed using a sport-specific time frame or method, which may limit the practical applicability of the findings to real wrestling performance. Third, upper-body neuromuscular and wrestling-specific performance outcomes were not evaluated and should be included in future investigations. Although the FR protocol targeted multiple regions, certain exercises required upper-limb support and core stabilization, which involves isometric contractions that may transiently alter cardiovascular and autonomic activation. Such engagement may have contributed to minor variations in HRV and BP responses, although these effects were likely brief relative to the systemic autonomic alterations induced by the preceding match simulation. Fourth, HRV measurements were performed only in the supine position, while the addition of seated measurements may provide further insight into cardiac-autonomic recovery [5, 64]. Fifth, the study sample consisted of only national-level male wrestlers. Thus, future research in female wrestlers is needed to examine potential sex differences. Finally, the absence of significant condition × time interactions, combined with standardized differences (Hedges’ g) favoring FR (and to a lesser extent SS) for HRV at 10 min post-recovery, indicates that studies with larger samples are needed to determine whether these modalities confer reliable recovery advantages over PR.

## Conclusion

FR, SS, and PR showed similar recovery patterns after a maximal-intensity wrestling match, and none of the methods fully restored autonomic or neuromuscular function within the short recovery period. Although Hedges’ *g* findings suggest modestly greater cardiac-autonomic recovery at 10 min post-recovery, particularly for FR versus PR, these magnitude-based observations remain exploratory in the absence of statistical significance. Collectively, the present findings do not provide conclusive evidence to recommend FR or SS as superior post-match recovery modalities over PR in high-level male wrestlers.

## Supplementary Information

Supplementary Material 1.

## Data Availability

Data are available for research purposes upon reasonable request to the corresponding author.

## Abbreviations

BP: Brachial blood pressure
CI: Confidence intervals
CMJ: Countermovement jump
DBP: Diastolic blood pressure
ES: Effect size
FR: Foam rolling
HRV: Heart rate variability
PR: Passive rest
RMSSD: Root mean square of successive RR-interval differences
SBP: Systolic blood pressure
SDNN: Standard deviation of normal R–R intervals
SS: Static stretching
TOPC: Turkey Olympic Preparation Center
